# Supplementary material for: Seroprevalence, Prevalence, and Genomic Surveillance: Monitoring the Initial Phases of the SARS-CoV-2 Pandemic in Betim, Brazil
Source: Front Microbiol. 2022 Feb 7;13:799713. doi: 10.3389/fmicb.2022.799713 (PMC8859412; doi:10.3389/fmicb.2022.799713)
Supplement: Supplementary file 1 [file Data_Sheet_1.PDF]

Table S1: Sequencing statistics.

ID	Raw	Paired_filtered	Unpaired_filtered	Mapped	Average_depth	Coverage
LBIb1520	790922	614298	86891	680628	2670.83	0.998562065
LBIb1756	895726	698392	96497	756729	2972.99	0.998495185
LBIb1853	595660	110010	221855	312410	996.48	0.949438202
LBIb1521	970750	684820	140045	787387	3056	0.998361423
LBIb1367	898558	520784	172820	321874	1262.4	0.998327983
LBIb1730	604232	162584	211009	118340	449543	0.992409042
LBIb1834	713926	506904	100255	519339	2027.59	0.998361423
LBIb1338	443152	218098	99202	144518	562251	0.996956929
LBIb1155	587778	276476	135914	60791	239	0.973481808
LBIb1128	589594	280462	142500	64184	250	0.976056715
LBIb1957	1096024	975312	32986	854776	4308.04	0.877407705
LBIb0013	288132	240058	3544	233727	1080.49	0.991873997
LBIb2769	555052	486824	11649	250565	1210.8	0.806046014
LBIb2626	312768	260432	8929	210031	1129.37	0.943151418
LBIb2405	931682	830622	12449	812147	4070.16	0.993077849
LBIb2427	245794	200684	9298	102113	532031	0.790730337
LBIb2421	970744	866408	19461	797757	3964.35	0.935995185
LBIb2791	410522	373574	5169	370680	2036.86	0.990001338
LBIb2224	2768498	2618126	30325	2600398	13991.7	0.993010968
LBIb2933	680422	622700	7978	613536	3254.58	0.973147405
LBIb2624	556428	511752	7062	507512	2796	0.9179374
LBIb2621	802890	747922	10676	717957	4050.13	0.977962814
LBIb2905	1291410	1067698	50017	1000586	5093.82	0.955557785
LBIb3231	3115324	2888612	39358	2854534	15183.4	0.99705725
LBIb2256	949972	892682	10741	883323	4972.17	0.97950107
LBIb2808	834356	739114	14535	723412	3741.48	0.943820225
LBIb2964	822522	722510	12942	314131	1762.05	0.934691011
LBIb3167	1060064	972588	12679	965926	5015.61	0.993846977
LBIb2674	1398626	1279472	28774	1236914	6484.77	0.9941145
LBIb1905	2295810	1661904	174732	1370724	6910.15	0.97752809
LBIb1806	2111520	1906066	61989	1885222	10040.7	0.989332531
LBIb1706	2368546	1554024	290025	1170912	6275.13	0.976290797
LBIb2296	2508976	1884492	202645	1600481	7961.24	0.991372392
LBIb2892	2369302	2159526	63471	2149739	11672.1	0.991673355
LBIb2494	2285012	2088736	52678	1919668	10072	0.986824505
