# Supplementary material for: Seroprevalence, Prevalence, and Genomic Surveillance: Monitoring the Initial Phases of the SARS-CoV-2 Pandemic in Betim, Brazil
Source: Front Microbiol. 2022 Feb 7;13:799713. doi: 10.3389/fmicb.2022.799713 (PMC8859412; doi:10.3389/fmicb.2022.799713)
Supplement: Supplementary file 2 [file Data_Sheet_2.PDF]

Table S2: Association of clinical and epidemiological data with a positive test (serological or molecular). Bolded p values indicate p < 0.05.

Variable	Level	Positive	Negative	p-value
	First	5 (4.7%)	1074 (34.3%)	
	Second	29 (27.4%)	1051 (33.5%)	
Survey	Third	72 (67.9%)	1008 (32.2%)	**< 0.0001**
	Alterosas	18 (17.0%)	616 (19.7%)	
	Citrolândia	4 (3.8%)	215 (6.9%)	
	Icaivera	0 (0.0%)	62 (2.0%)	
	Imbiruçu	32 (30.2%)	533 (17.0%)	
	Norte	11 (10.4%)	322 (10.3%)	
	Petrovale	0 (0.0%)	41 (1.3%)	
	PTB	8 (7.5%)	282 (9.0%)	
	Sede	15 (14.2%)	568 (18.1%)	
	Terezópolis	17 (16.0%)	302 (9.6%)	
Administrative Regions	Vianópolis	1 (0.9%)	192 (6.1%)	**0.002424**
Sex	Female	69 (65.1%)	1559 (49.8%)	**0.002642**
	Male	37 (34.9%)	1574 (50.2%)	
	0-5	3 (2.8%)	214 (6.8%)	
	06-19	15 (14.2%)	635 (20.3%)	
	20-39	42 (39.6%)	1025 (32.7%)	
	40-59	31 (29.2%)	840 (26.8%)	
Age range	Above60	15 (14.2%)	419 (13.4%)	0.190538
	No	106 (100.0%)	3119 (99.6%)	
International travel	Yes	0 (0.0%)	14 (0.4%)	1
	No	88 (83.0%)	2927 (93.4%)	
Fever	Yes	18 (17.0%)	206 (6.6%)	**0.000075**
	No	73 (68.9%)	2518 (80.4%)	
Cough	Yes	33 (31.1%)	615 (19.6%)	**0.005304**
	No	77 (72.6%)	2765 (88.3%)	
Sore throat	Yes	29 (27.4%)	368 (11.7%)	**0.000003**
	No	96 (90.6%)	3002 (95.8%)	
Dyspnoea	Yes	10 (9.4%)	131 (4.2%)	**0.018051**
	No	72 (67.9%)	2883 (92.0%)	
Myalgia	Yes	34 (32.1%)	250 (8.0%)	**< 0.0001**
	No	70 (66.0%)	2452 (78.3%)	
Rhinorrhea	Yes	36 (34.0%)	681 (21.7%)	**0.004198**
	No	90 (84.9%)	2961 (94.5%)	
Respiratory discomfort	Yes	16 (15.1%)	172 (5.5%)	**0.000079**
	No	94 (88.7%)	3025 (96.6%)	
Nausea/ vomit	Yes	12 (11.3%)	108 (3.4%)	**0.000075**
	No	50 (47.2%)	2399 (76.6%)	
Headache	Yes	56 (52.8%)	734 (23.4%)	**< 0.0001**
	No	83 (78.3%)	2968 (94.7%)	
Prostration	Yes	23 (21.7%)	165 (5.3%)	**< 0.0001**
	No	95 (89.6%)	2933 (93.6%)	
Diarrhea	Yes	11 (10.4%)	200 (6.4%)	0.150267
	No	105 (99.1%)	3102 (99.0%)	
Conjunctivitis	Yes	1 (0.9%)	31 (1.0%)	1
	No	87 (82.1%)	3051 (97.4%)	
Ageusia/ anosmia	Yes	19 (17.9%)	82 (2.6%)	**< 0.0001**
	No	102 (96.2%)	3081 (98.3%)	
Loss of voice	Yes	4 (3.8%)	52 (1.7%)	0.206495
	No	104 (98.1%)	3105 (99.1%)	
Pneumopathy	Yes	2 (1.9%)	28 (0.9%)	0.593166
	No	106 (100.0%)	3094 (98.8%)	
Chronic neurological disease	Yes	0 (0.0%)	39 (1.2%)	0.482095
	No	104 (98.1%)	3107 (99.2%)	
Pregnant	Yes	2 (1.9%)	26 (0.8%)	0.533509
	No	105 (99.1%)	3125 (99.7%)	
Postpartum	Yes	1 (0.9%)	8 (0.3%)	0.699884
	No	106 (100.0%)	3037 (96.9%)	
Chronic cardiovascular disease	Yes	0 (0.0%)	96 (3.1%)	0.123958
	No	103 (97.2%)	3086 (98.5%)	
Chronic kidney disease	Yes	3 (2.8%)	47 (1.5%)	0.489013
	No	96 (90.6%)	3038 (97.0%)	
Obesity	Yes	10 (9.4%)	95 (3.0%)	**0.000721**
Asthma	No	102 (96.2%)	2964 (94.6%)	0.609912

	Yes	4 (3.8%)	169 (5.4%)	
Immunodepression	No	104 (98.1%)	3113 (99.4%)	0.348298
	Yes	2 (1.9%)	20 (0.6%)	
Chronic liver disease	No	106 (100.0%)	3118 (99.5%)	1
	Yes	0 (0.0%)	15 (0.5%)	
Diabetes	No	100 (94.3%)	2911 (92.9%)	0.71047
	Yes	6 (5.7%)	222 (7.1%)	
Hypertension	No	91 (85.8%)	2585 (82.5%)	0.445923
	Yes	15 (14.2%)	548 (17.5%)	
Transplanted	No	106 (100.0%)	3129 (99.9%)	1
	Yes	0 (0.0%)	4 (0.1%)	
Cancer	No	106 (100.0%)	3110 (99.3%)	0.766303
	Yes	0 (0.0%)	23 (0.7%)	
Any comorbidity	No	73 (68.9%)	2211 (70.6%)	0.787174
	Yes	33 (31.1%)	922 (29.4%)	
Sought health assistance	Hospital	8 (7.5%)	130 (4.1%)	**0.003285**
	None	81 (76.4%)	2764 (88.2%)	
	Basic Health Unit	8 (7.5%)	121 (3.9%)	
	Emergency Care Unit	9 (8.5%)	118 (3.8%)	
Admitted in health institution	No	103 (97.2%)	3098 (98.9%)	0.249183
	Yes	3 (2.8%)	35 (1.1%)	
Household contact with symptomatic person	No	71 (67.0%)	2528 (80.7%)	**0.000774**
	Yes	35 (33.0%)	605 (19.3%)	
